# Molecular Basis for Modulation of the p53 Target Selectivity by KLF4

**DOI:** 10.1371/journal.pone.0048252

**Published:** 2012-10-30

**Authors:** Tobias Brandt, Fiona M. Townsley, Daniel P. Teufel, Stefan M. V. Freund, Dmitry B. Veprintsev

**Affiliations:** 1 Medical Research Council Laboratory of Molecular Biology, Cambridge, United Kingdom; 2 Max Planck Institute of Biophysics, Frankfurt am Main, Germany; 3 Laboratory of Biomolecular Research, Paul Scherrer Institut, Villigen PSI, Switzerland; 4 Department of Biology, ETH Zürich, Zürich, Switzerland; Institut Pasteur, France

## Abstract

The tumour suppressor p53 controls transcription of various genes involved in apoptosis, cell-cycle arrest, DNA repair and metabolism. However, its DNA-recognition specificity is not nearly sufficient to explain binding to specific locations *in vivo*. Here, we present evidence that KLF4 increases the DNA-binding affinity of p53 through the formation of a loosely arranged ternary complex on DNA. This effect depends on the distance between the response elements of KLF4 and p53. Using nuclear magnetic resonance and fluorescence techniques, we found that the amino-terminal domain of p53 interacts with the KLF4 zinc fingers and mapped the interaction site. The strength of this interaction was increased by phosphorylation of the p53 N-terminus, particularly on residues associated with regulation of cell-cycle arrest genes. Taken together, the cooperative binding of KLF4 and p53 to DNA exemplifies a regulatory mechanism that contributes to p53 target selectivity.

## Introduction

The tumour suppressor p53 is at the centre of a large network responsible for the transcription of genes involved in apoptosis, senescence and cell-cycle arrest. Its function is to maintain genomic integrity upon stress. It is not surprising that in approximately half of all characterised tumours, p53 is mutated and dysfunctional [1]. In addition, p53 also controls the transcription of genes involved in a variety of cell survival processes such as DNA repair, metabolism regulation or embryo implantation [2], [3]. Sequence specific recognition of DNA response elements is a key to correct functioning of p53 (reviewed in [4]). However, increasing biophysical evidence suggest that DNA-binding specificity of the transcription factors themselves is not nearly sufficient to explain their binding to so few specific locations identified in the whole genome [5], [6], [7]. The p53 homologs p63 and p73 also recognise the same response elements [8], but carry out functions distinct from p53 [9]. The situation is further complicated by the fact that the genome contains a very large number of putative p53/p63/p73 response elements [6], most of which are not necessarily involved in transcription regulation.

**Figure 1:**
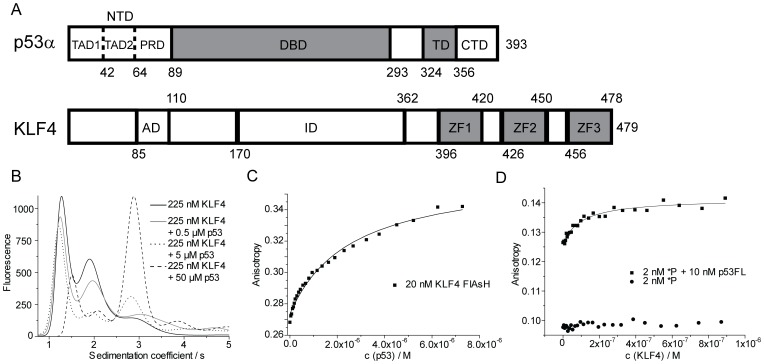
KLF4 and p53 directly interact. A: Domain structure of p53 and KLF4. Folded domains are shown in grey. p53 comprises an N-terminal domain (NTD) consisting of the transactivation domains 1 and 2 (TAD1, TAD2) and the proline-rich domain (PRD), a DNA-binding domain (DBD), a tetramerisation domain (TD), and a C-terminal domain (CTD). KLF4 domain boundaries for the transcriptional activation domain (AD) and inhibitory domain (ID) are approximate. Three zinc fingers (ZF) are encoded at the C-terminus. B: Normalised sedimentation coefficient distributions measured by FDSV-AUC. 225 nM FlAsH-labelled KLF4 in the absence (black) and in presence of 0.5 µM (grey), 5 µM (dotted line) and 50 µM (dashed line) unlabelled p53. Engineered, neutrally stabilised quadruple mutant M133L/V203A/N239Y/N268D p53 was used throughout this study. C: Direct interaction between p53 and KLF4. Fluorescence anisotropy titration at 110 mM total ionic strength with FlAsH-labelled KLF4 as probe and p53 as titrant. D: Fluorescence anisotropy titrations with a labelled p53 RE (*P). No binding is observed if KLF4 is titrated into a DNA-only solution (circles). If a *P-p53 complex is used as a probe (note higher anisotropy value, squares, FA285 buffer), a binding event can be observed.

A number of regulatory mechanisms have been suggested that provide transcriptional selectivity and the desired cellular response, such as cell death or survival [2], [10]. Such regulatory mechanisms involve the DNA-binding properties of p53, the state of chromatin, the concentration of p53 in the nucleus, p53 protein-interactions and post-translational modifications. All domains of p53 (Figure 1A) are involved in some of these regulatory mechanisms. p53 binds DNA as a dimer of dimers [11], [12]. Specific contacts are made by the four p53 DNA-binding domains (p53DBD) to the 20 base pair (bp) canonical consensus response element (RE) defined as two repeats of the RRRCWWGYYY (R = A/G, W = A/T, Y = C/T) decamer [13]. Moreover, it has become evident that p53 can also bind promoters which deviate from this sequence or are only composed of half or three-quarter canonical REs [4], [6], allowing p53 to bind a more diverse range of targets. In addition to sequence-specific DNA binding, non-specific contact is made to DNA by the p53 C-terminal domain (p53CTD). Thus, p53 is able to slide along DNA in a search for its REs, providing a more efficient recognition mechanism than random diffusion and dissociation [14], [15]. Another proposed mechanism involves conformational changes in the L1 loop of the DNA binding domain [16]. Recently, it has been shown that p53 adopts different conformations when bound to DNA via its CTD or via its DBD [17], or that the acetylation of the DNA-recognition domain leads to an increase in its DNA-binding specificity at lower ionic strength [18]. However, it is not clear how the proposed mechanisms increase the selectivity of p53 for a particular sub-set of available binding sites, as well as its specificity over p63 and p73.

Several transcription factors are known to modulate p53 transcriptional activity [4]. The transcription factor gut-enriched Krüppel-like factor 4 (KLF4, Figure 1A) is involved in cell-cycle progression and proliferation. It has been reported to be either a tumour suppressor or an oncogene, depending on the context [19]. Like p53, KLF4 is necessary to mediate G_1_/S phase arrest [20]. A link between KLF4 and the p53 apoptosis/cell-cycle arrest decision has been established. KLF4 is activated following mild DNA damage and promotes cell-cycle arrest, but is repressed upon severe DNA damage leading to cell death [21]. Cancer cells switch their p53-response under γ-irradiation from apoptosis towards cell-cycle arrest upon KLF4 expression [22]. Further, KLF4 induces transcription on the *p21* ^WAF1/Cip1^ promoter synergistically with p53, and both proteins co-immunoprecipitate [23]. A ChIP analysis has shown that KLF4 and p53 are bound to the *p21* ^WAF1/Cip1^ and the *BAX* promoters [22].

We hypothesised that the interaction with another transcription factor (TF) may enhance p53 target selectivity via ternary complex formation on DNA. Most promoters contain REs for more than one TF, possibly permitting the cooperative DNA-binding of two interacting TFs. Thus, transcriptional selectivity may be achieved by the combination of appropriate REs of more than one TF within a promoter region. Here, we use biophysical methods to characterise the interaction between KLF4 and p53 and study its effect on p53 DNA-binding. We show that p53 and KLF4 directly interact, mapped their interaction sites using nuclear magnetic resonance spectroscopy, and demonstrated that phosphorylation of p53 enhances this interaction. We directly measured the effect of cooperative binding of two transcription factors to DNA using a fluorescence anisotropy assay. Strikingly, KLF4 increases the DNA-binding affinity of p53, and the increase is dependent on the distance between the p53 and KLF4 response elements. The concerted cooperative action of two transcription factors on binding their respective response elements exemplifies a regulatory mechanism that enhances transcriptional target selectivity.

## Materials and Methods

### Gene, Protein, and DNA Preparation

Standard protocols were used for the preparation of plasmids, proteins and fluorescently labelled DNA. We have used an engineered neutrally stabilised quadruple mutant (M133L/V203A/N239Y/N268D) of p53 as it is more suitable for extended biophysical studies than the wild type protein [24], [25], [26]. A detailed description can be found in the supplementary information (file Text S1).

### Fluorescence Anisotropy Measurements

Fluorescence anisotropy was recorded on a Cary Eclipse spectrometer (Varian) equipped with a titrator (Hamilton). Excitation and emission wavelengths were 480 nm and 530 nm, respectively. Solutions containing labelled DNA (2–4 nM) only, labelled DNA (2–4 nM) and unlabelled protein (40–4000 nM), or labelled KLF4-FlAsH (20 nM) were stirred inside the cuvettes. Into this fluorescent solution a second protein was titrated. The concentration of this titrant varied between 100 nM and 10 µM depending on the affinity to the studied DNA/DNA-protein complex in order to obtain high quality titration curves. All experiments were performed at 20°C in 5 mM DTT, 25 mM NaP_i_ (pH 7.2), 10% glycerol, 0.2 mg/mL BSA (Sigma) and 50, 100, 150 or 225 mM NaCl, corresponding to a total ionic strength of 110, 160, 210 or 285 mM (FA110/160/210/285 buffer). Fluorescence intensities were measured after an equilibration time of 60 s after each injection. Data were analysed using laboratory software as has been described before [27].

Titrations with coumarin labelled p53 peptides were done as previously described [28]. 200 nM solutions of peptide in 50 mM NaCl, 25 mM NaPi pH 7.2, 10% (v/v) glycerol and 5 mM DTT were used. Titrant solution concentration was 100–150 µM. Excitation and emission wavelengths were 328 nm and 392 nm, respectively.

### Analytical Ultracentrifugation

We used XL-I analytical ultracentrifuges (Beckman) equipped with absorbance or fluorescence detection systems (AVIV Biomedical). Sedimentation velocity experiments with unlabelled KLF4 or FlAsH-tagged protein were done in 150 mM NaCl, 25 mM phosphate (pH 7.2), 10% glycerol, BSA (0.2 mg/mL, not used with absorbance detection) and 1 mM β-mercaptoethanol at 10°C as described previously at 45–50 k rpm [8]. Concentrations of 5 µM unlabelled KLF4 and 75–225 nM KLF4-FlAsH were used. 25–50 nM labelled DNA and 1 µM unlabelled KLF4 were used in experiments detecting protein-DNA complexes. Buffer density and viscosity were calculated using the SEDNTERP software. Data analysis to obtain sedimentation coefficient traces was done with the SEDFIT software [29].

### NMR

Samples were dialysed twice overnight at 4°C into 25 mM NaP_i_ buffer at pH 7.2, with the addition of 150 mM NaCl and 5 mM DTT. 35 µL ^2^H_2_O (Sigma) were added to 500 µL sample.

All experiments were acquired at 20°C on Bruker DRX-600 or Avance 700 spectrometers. For binding experiments, ^1^H, ^15^N-HSQC spectra were obtained with ^15^N-labelled protein in presence and absence of ligand. To obtain high quality 2D correlation maps, the p53 TC construct was ^2^D, ^15^N-labelled and relaxation-optimised ^1^H, ^15^N TROSY sequences replaced standard HSQC experiments. Protein concentrations were in the region of 50–100 µM, whereas the peptide ligands were added in 2–4-fold excess. The backbone ^1^H,^15^N and ^13^C assignments for KLF4 (367–479) (300 µM) were obtained using a standard set of triple resonance experiments. 90% of all resonances were assigned unambiguously. A subset of low intensity resonances suggests the presence of a population of residues neighbouring prolines in *cis*-conformation. All data were processed in Topspin (Bruker, Karlsruhe) and analysed in Sparky [30]. An initial automated backbone assignment was obtained with MARS [31], and then manually completed with in-house perl scripts.

### Bioinformatics

The human genome sequence (hg19,GRCh37), masked for repeats, was analysed for the presence of putative p53 and KLF4 response elements using p53BindingPredictor software [6]. We used previously reported positional weight matrixes for KLF4 [32] and p53 [6].

## Results

### Characterisation of KLF4 and p53

We expressed recombinant full-length p53 and KLF4 (Figure 1A). We used a thermo-stable quadruple mutant (M133L/V203A/N239Y/N268D) of p53 (from here on referred to as p53) which has previously been extensively studied and biophysically characterised and is stable for the duration of our measurements [6], [8], [25], [33], [34]. Here, we determined the oligomeric state of KLF4 with and without DNA and characterised its long-term stability. Using analytical ultra-centrifugation (AUC) we found that full-length KLF4 was monomeric in solution and that the sample was homogenous and did not aggregate as judged by the presence of only one peak in the sedimentation coefficient distribution profile (Figure S1A and B). Furthermore, NMR experiments (see below) showed that the three carboxy-terminal zinc finger domains remain folded over the length of a 3D-NMR experiment at 20°C (several days). The remaining parts of the protein were, as predicted by sequence analysis, natively unfolded. Using AUC and fluorescence anisotropy titrations, we showed that the monomer of KLF4 binds DNA in a sequence-specific manner (Figure S1B and C, Tables S1 and S2). Additional details of AUC and titration experiments are presented within the supplementary information results section (file Text S2).

### Direct Interaction between KLF4 and p53

Several studies have been focussed on the *in vivo* effect of KLF4 on p53-mediated transcription [20], [21], [22], [23]. In order to test the hypothesis of concerted DNA-binding by two transcription factors, we carried out experiments to establish whether KLF4 and p53 interact directly.

Firstly, we established that the full-length proteins interact, using fluorescence detection analytical ultracentrifugation (FD-AUC) and FlAsH-labelled KLF4 as a probe. The sedimentation profile of KLF4-FlAsH showed a peak at S = 1.3, corresponding to monomeric KLF4, and a second peak at S = 2.0. Upon addition of unlabelled p53 to labelled KLF4 a third peak at S = 3.0 appeared, indicating formation of a KLF4-p53 complex (Figure 1B). The peak at S = 2.0 can be attributed to a cross-link via the cysteines of the FlAsH-tag multimer of KLF4, because it was not observed for non-labelled KLF4 (Figure S1A).

Secondly, KLF4-FlAsH was used as a fluorescent probe in fluorescence anisotropy titrations at 110 mM total ionic strength (Figure 1C). The incremental addition of p53 yielded a *K*_d_ of 2.6 µM, confirming the direct interaction between the two proteins. In order to determine whether the binding affinity of KLF4 towards p53 is altered if p53 is DNA-bound, we used p53 in complex with its labelled RE (*P) as a probe (Figure 1C). Tight binding (*K*_d_ = 100 nM) between a pre-formed p53-DNA complex (5∶1) and KLF4 was observed at 285 mM ionic strength, whereas KLF4 did not bind free *P. The affinity increase in comparison to unbound p53 is striking, especially considering the high ionic strength used which was necessary to prevent KLF4 binding to free *P. Hence, the KLF4-p53 interaction is stimulated when p53 is in complex with DNA.

### Mapping the Interaction between p53 and KLF4

**Figure 2:**
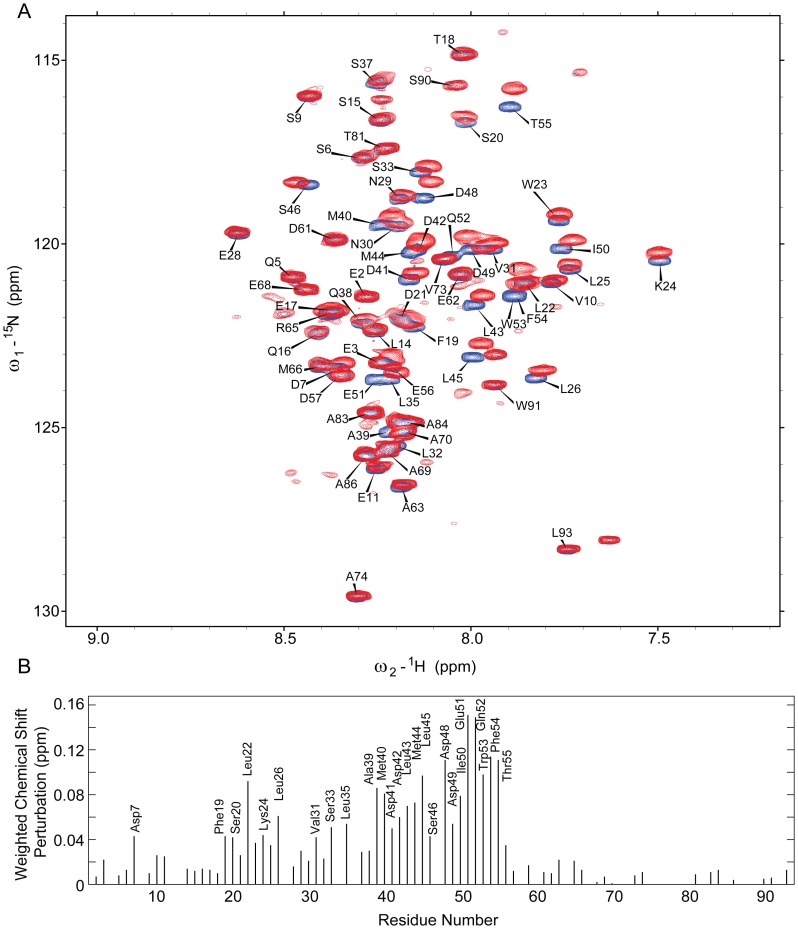
Identification of interacting residues in p53 by NMR. A: Overlay of 2D ^1^H, ^15^N-HSQC NMR spectra for p53 NTD in the absence (blue) and presence of 135 µM KLF4 271–479 (red). B: Weighted chemical shift perturbation map of ^15^N-p53NTD upon addition of KLF4 271–479. No data were obtained for prolines and residues with no assigned resonances.

Nuclear magnetic resonance (NMR) spectroscopy was used to characterise the interaction between KLF4 and p53 at single residue resolution. Based on reported backbone assignments [35], ^1^H, ^15^N-heteronuclear single quantum coherence (HSQC) experiments were recorded with ^15^N-p53NTD (1–93). Spectra in the absence and presence of KLF4 (271–479) revealed significant chemical shift perturbations (Figure 2A) and enabled us to identify residues of p53NTD which were involved in the interface with KLF4 (Figure 2B). The residues exhibiting the highest chemical shift perturbations clustered in the TAD2 region of p53 (transactivation domain, residues 40–65). Furthermore, several residues within the TAD1 region of p53 (residues 1–39) were also affected by the addition of KLF4. No binding to the proline-rich region (residues 66–93) was detected. Further experiments with ^15^N-p53DBD (94–292) and ^2^H, ^15^N-p53TC (313–393) showed no binding to KLF4 (271–479) (Figure S2), indicating that the p53 N-terminus is the sole interaction site with KLF4.

**Figure 3:**
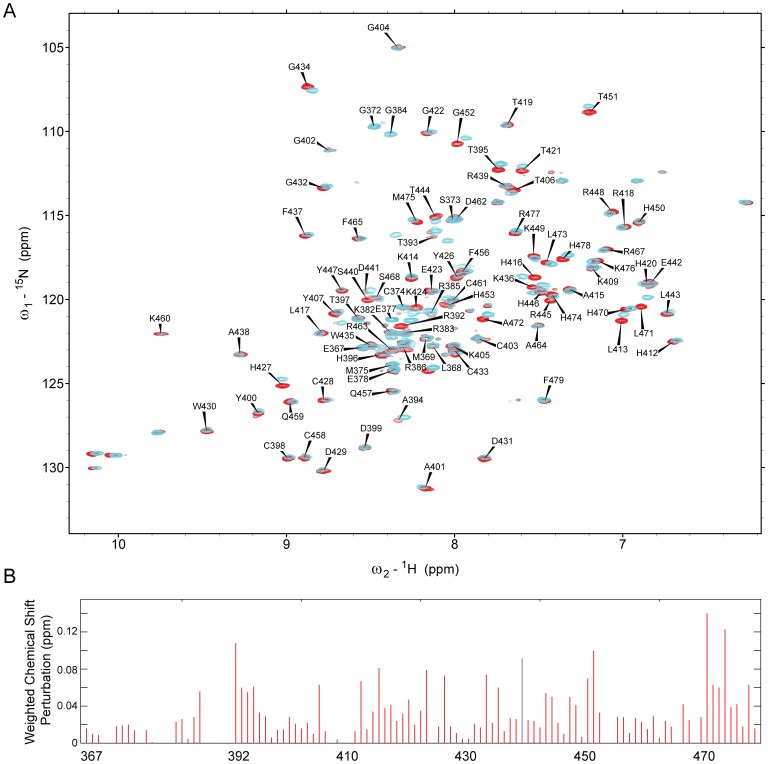
Identification of the binding interface in KLF4 367–479. A: 2D ^1^H,^15^N-HSQC spectrum for KLF4 (367–479) (red), labelled with resonance assignments, overlaid with a spectrum observed in the presence of 300 µM p53 (1–57) pT55 as a ligand (cyan). B: Chemical shift perturbations of KLF4 (367–479) residues upon addition of p53 (1–57) pT55. No signal was observed for Ser440 (blue) in the presence of p53.

To identify the p53NTD binding site on KLF4, ^15^N-HSQC experiments were performed using a ^15^N-labelled KLF4 (367–479) construct, in the absence and presence of p53 (1–57) as a ligand. The 2D ^15^N-HSQC spectrum of KLF4 (367–479) was indicative of a structured domain as a result of the presence of three zinc finger domains (Figure 3A), but also contained a subset of resonances at a random coil chemical shift position, characteristic for unfolded residues. Addition of p53 (1–57) or p53 (1–57)pT55 resulted in significant chemical shift perturbations (Figure 3A and B). Interestingly, phosphorylation of T55 in the p53 (1–57) construct lead to additional chemical shift perturbations not observed for the non-phosphorylated p53 (1–57).

Backbone assignments for KLF4 residues were based on a ^13^C, ^15^N-labelled KLF4 (367–479) sample and standard triple-resonance 3D experiments (Table S3). Upon binding to p53NTD (1–93), p53 (1–57) and p53 (1–57) pT55, resonances of KLF4 residues R392, G452, L471, and H474 showed the largest perturbations (Figure 3A and B, Figure S3). The signal for S440 disappeared which may be a sign for involvement in binding. Separate binding sites were apparent on all three zinc-fingers. Residues R386–T395 constituted an additional binding site in an unfolded region at the N-terminus of the first zinc finger. T406 was included into this binding site, because of its proximity in the crystal structure [36]. This binding site is essential for cooperative DNA binding of KLF4 and p53 (see below). Large chemical shift perturbations for residues K424 and H427 in KLF4 were only observed in the presence of the T55 phosphorylation on the p53 (1–57) peptide. Interestingly, in the crystal structure of the DNA-bound KLF4 [36] the side chain of K424 binds the phosphate backbone of DNA.

KLF4 binds exclusively to the N-terminus of p53 via its C-terminal region, and no binding to ^15^N-KLF4 (367–479) was observed for p53TC (293–393) covering the linker region not tested before (Figure S4). Furthermore, no interaction with p53 was observed for ^15^N-KLF4 (271–390), the C-terminal part of the inhibitory domain, which is completely unfolded (Figure S5).

### Phosphorylated p53 Binds More Tightly to KLF4

Fluorescence anisotropy titrations were used to further characterise the interaction between KLF4 and the p53NTD based on N-terminal truncated constructs of KLF4 bound to coumarin-labelled p53NTD peptides p53 (1–57) and (38–57) (Figure S6). KLF4 (271–479) and KLF4 (367–479) bind p53 (1–57) with a *K*_d_ of 30 µM. No binding was detected between p53 (1–57) and KLF4 (391–479), as well as p53 (38–57) and KLF4 (271–479) or KLF4 (391–479). We can conclude, that residues within p53 (1–57) and KLF4 (367–479) are necessary for the p53-KLF4 interaction.

Further, we studied the effect of phosphorylation at residues S15, T18, S20, S33, S37, S46, and T55 on p53 binding to KLF4 (367–479) (Table 1). Phosphorylation at S15, T18, S20, S33, and S37 in the TAD1 region increased the affinity of KLF4 towards p53 three to four times, whereas phosphorylation at S46 and T55 within TAD2 increased the affinity about 8.5 times. A p53 (1–57) peptide phosphorylated at all seven serine and threonine positions showed a 25-fold increase in affinity to KLF4.

**Table 1:** Binding of labelled N-terminal p53 peptides to KLF4 367–479.

Labelled peptide	*K*_d_ (KLF4 367–479)/µM*	*Enhancement ratio*
p53 1–57	30±11	1
p53 1–57 pS15	9.5±2.1	3
p53 1–57 pT18	6.9±1.8	4
p53 1–57 pS20	8.1±1.6	4
p53 1–57 pS33	6.6±2.1	4
p53 1–57 pS37	8.5±1.9	4
p53 1–57 pS46	3.5±0.9	8
p53 1–57 pT55	3.6±1.1	8
p53 10–57 hepta P	1.2±0.3	25

* Experiments were carried out at low ionic strength (110 mM) in order to reliably measure the binding constant. NMR experiments confirmed binding at physiological ionic strength.

### KLF4 Enhances Specific DNA-binding Affinity of p53 *in vitro*

**Figure 4:**
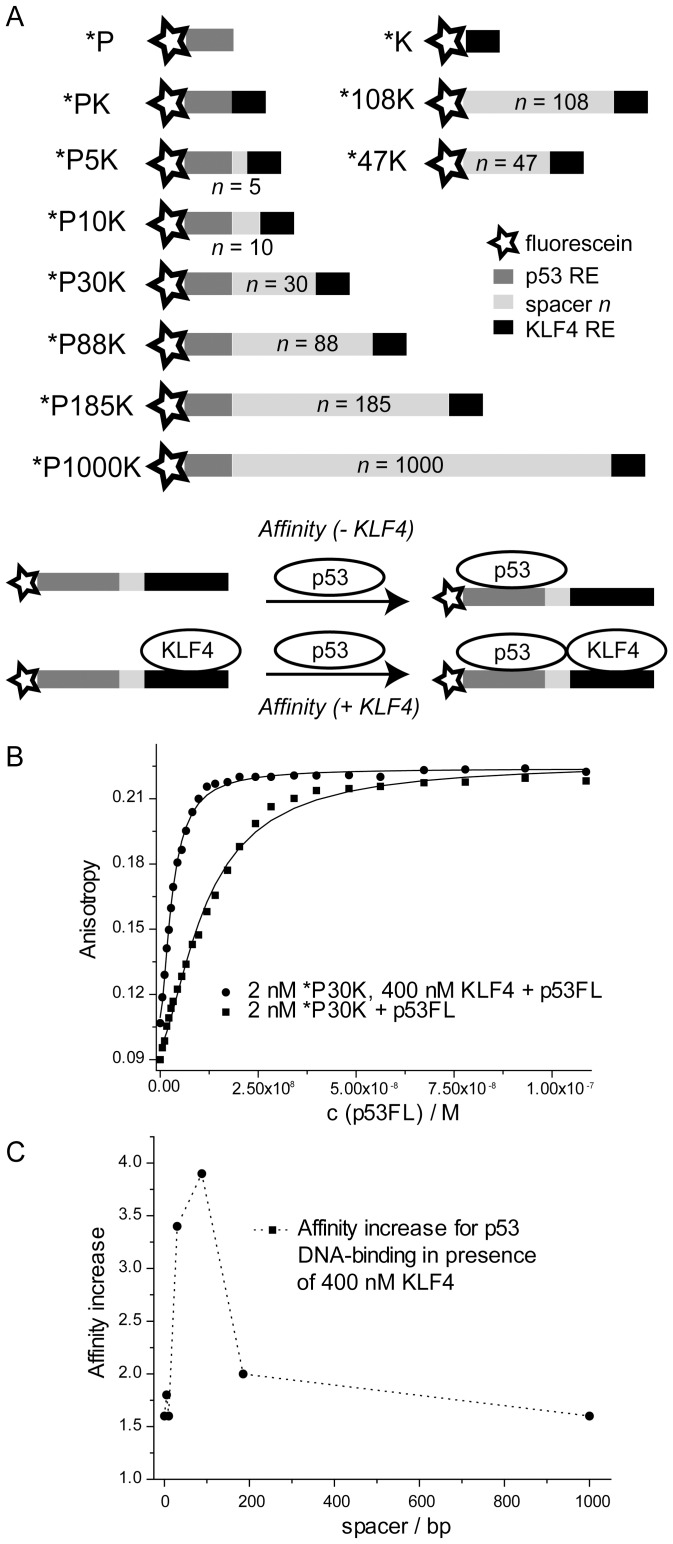
KLF4 enhances the DNA-binding affinity of p53. A: DNA constructs generated and principle of cooperative fluorescence anisotropy titrations. Fluorescein (*, star), a p53 RE (*P*, dark grey), a spacer (*n*, light grey), and a KLF4 RE (*K*, black) compose the labelled DNA. The affinity of p53 towards DNA is measured in the presence (+ KLF4) and absence (- KLF4) of KLF4. B: Example p53 titration data using *P30K as DNA in the presence (circles) and absence (squares) of KLF4 in FA285 buffer. C: Normalised p53 DNA-binding affinity in the presence of 400 nM KLF4 as a function of the distance between the p53 RE and the KLF4 RE.

Having established that and how KLF4 and p53 interact, we analysed which effect the protein-protein interaction might have on p53 DNA-binding properties. To assess the ability of KLF4 and p53 to bind DNA cooperatively, we developed a fluorescence anisotropy titration assay for which we generated various fluorescent DNA constructs (Figure 4A, Table S1). These constructs contained a 5′ fluorescent label (*), followed by a p53 RE (P), a spacer of variable length *n*, and a KLF4 RE (K). Several control constructs lacked either the p53 or the KLF4 RE. Important control experiments showed that KLF4 did not bind the p53 RE (*P) and vice versa (Tables 2 and S2). With fluorescence anisotropy titrations the binding of p53 to short oligonucleotides can be reliably detected [6], [8], [12], [27], [37]. We observed that, in general, p53 showed a higher affinity to elongated DNA sequences in comparison to *P. This may have several reasons. Firstly, non-specific binding of the p53 carboxy-terminal domain is not possible to the short DNA-sequences, but is likely to contribute to the overall binding if the DNA molecule is sufficiently long. However, at 285 mM ionic strength this effect has been shown to be relatively small [18]. Secondly, p53 binds many DNA sequences weakly such as those which comprise only a half or three-quarter binding site [4], and which are present in most DNA sequences. Lastly, the oligonucleotides span a wide range of lengths and were produced either by PCR or by chemical synthesis, possibly contributing to changes in the absolute affinity values. While these experimental limitations may not be avoided, the important parameter that reflects the impact of KLF4 presence on the binding of p53 to DNA is the relative increase in the affinity for DNA of a given length. This parameter is not affected by the change of the absolute affinity of p53 for DNA in the absence of KLF4.

**Table 2:** Binding of p53 to DNA in dependence of added KLF4 and spacing between cognate binding sites.

DNA	KLF4 construct	*c* (KLF4)/nM	*K*_d_ ± SD/nM&	*Enhancement ratio*
*P	FL	0	45±7	–
		200	36±6	1.2
		1000	40	1.1
*K	–	–	n.b.	–
*PK	FL	0	8.1±1.7	–
		400	5.2±0.6	1.6
*P5K	FL	0	8.1±1.3	–
		400	4.6±0.7	1.8
*P10K	FL	0	9.5±1.8	–
		400	5.9±0.7	1.6
*P30K	FL	0	12±1.9	–
		400	3.5±0.4	3.4
*P88K	FL	0	3.9±0.7	–
		400	1.0±0.3	3.9
*P185K	FL	0	4.0±0.3	–
		400	2.0±0.7	2.0
*P1000K	FL	0	9.5±3.1	–
		400	5.9±2.0	1.6
*P30K	179–479	0	12±1.9	–
		400	4.4±0.6	2.7
*P30K	271–479	0	12±1.9	–
		400	4.4±0.6	2.7
*P30K	391–479	0	12	–
		400	10	1.2

& Measured by fluorescence anisotropy titrations in FA 285 buffer.

p53 was titrated into a solution of labelled DNA, in the presence or absence of KLF4 (Figure 4A and B). High concentrations (400 nM) of KLF4 were used in order to fully saturate the labelled DNA so that only one species (KLF4-bound DNA) was present in solution. If KLF4 was present, a higher starting anisotropy value was observed, indicating that KLF4 was bound to DNA. We found a significant increase in p53 binding affinity to fluorescein-labelled DNA encoding a p53 RE and a KLF4 RE in the presence of KLF4 (Table 2). By using spacers of different length, we assessed the effect of distance between the p53 and KLF4 REs. We found that the KLF4-dependent affinity increase of p53 to DNA is highest for DNA sequences with spacers of 30 (*P30K) and 88 (*P88K) base pairs (Table 2, Figure 4B and C). For shorter spacers of 0, 5, and 10 base pairs the effect is less pronounced, probably due to steric constraints imposed on the interaction sites. The increase in p53 affinity to DNA was also diminished if the spacer length was increased to hundreds of base pairs, suggesting an optimal distance being required for most efficient p53 RE binding. Very similar results were obtained for *PK, *P5K, *P10K and *P30K at an ionic strength of 285 mM (Table 2) and 210 mM (Table S5).

If a shortened KLF4 construct (391–479), which bound DNA equally well as full-length KLF4 (Table S2), was used, no effect on the DNA-binding of p53 was observed (Table 2). Additionally, corresponding control experiments with several other transcription factors, such as CP2, HSF1 and YY1, did not yield comparable increases in affinity (Table S4), confirming that the affinity enhancement of p53 to its RE can be specifically attributed to the presence of KLF4. Taken together, both control experiments showed that enhanced p53 DNA-binding affinity is not observed if a second transcription factor is bound to the same DNA but does not interact with p53.

Next, fluorescence anisotropy titrations, designed to ascertain ternary complex formation, were carried out, using *P DNA to which KLF4 did not bind (Table S2). No significant increase in affinity of p53 to *P was observed upon addition of KLF4 (Table 2). Hence, the mere presence of KLF4 in solution was not enough to enhance p53 binding to DNA. By consequence, an allosteric mechanism can be ruled out.

### KLF4 does not Affect Non-specific Binding of p53 to DNA

Further experiments were done with DNA containing weak non-canonical p53 REs. Several groups including us have previously shown that the non-specific interactions of p53 with DNA are mediated by several lysines of the C-terminal domain [12], [14], [38], [39]. This interaction is highly sensitive to variations of the ionic strength. In contrast, specific interactions are significantly less sensitive to the ionic strength. Consequently, at 210 mM ionic strength p53 predominantly binds DNA non-specifically via its C-terminus while at 285 mM ionic strength [17], non-specific interactions are suppressed and specific interactions are observed.

We assessed the effect of KLF4 on weak p53 binding sites at 285 mM ionic strength using the DNA sequence *108K which includes a weak three-quarter binding site (Figure S7A, Table S1). At this ionic strength, non-specific binding is significantly suppressed [18]. p53 bound weakly to this sequence (*K_d_* = 1200±420 nM). However, the affinity was remarkably increased by addition of 40 nM KLF4 (2-fold, *K_d_* = 680±93 nM), 100 nM KLF4 (5-fold, *K_d_* = 250 nM), and 400 nM KLF4 (8.5-fold, *K_d_* = 140±57 nM). We conclude, that KLF4 has the ability to transform weak p53 REs into more potent ones.

Secondly, we tested if KLF4 also affects non-specific p53-DNA interactions. These experiments were done at 210 mM ionic strength, using *47K DNA which also included a weak, three-quarter binding site. However, at this ionic strength, p53 strongly and dominantly interacts with DNA non-specifically via its carboxy-terminal domain [18]. The DNA-binding affinity of p53 towards *47K (25±5.2 nM) was not affected by the addition of 400 nM KLF4 (27±1.2 nM) (Figure S7B). Overall, these results indicate that the interaction between KLF4 and p53 enhances only the specific binding of p53 to DNA.

### The Extended Zinc-finger Region of KLF4 is Necessary for Increased DNA-binding of p53

We tested the ability of several deletion constructs of KLF4 (179–479, 271–479, 391–479) to enhance the DNA-binding affinity of p53. All KLF4 constructs bound DNA as well as the full-length protein (Table S2). Only the shortest KLF4 construct (391–479) was not sufficient to increase p53 DNA-binding affinity to the same extent as full-length KLF4 (Table 2). Elongation of this KLF4 construct towards the N-terminus (KLF4 367–479) restored the effect observed for full-length KLF4. A major p53-interaction site within KLF4 must therefore reside within residues 367–479, reflecting our NMR results (see above).

### *In silico* Identification of Co-localised KLF4 and p53 REs

**Figure 5:**
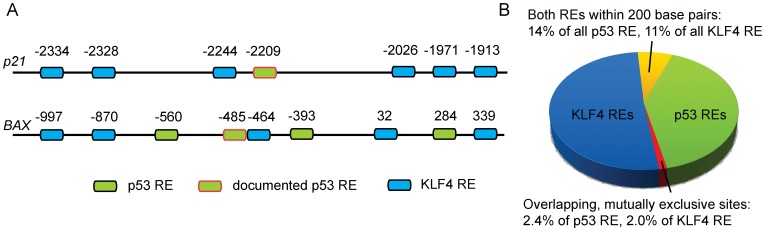
*In silico* analysis of the p53 and KLF4 response element co-localisation in the human genome. A: promoter regions of the *p21* ^WAF1/Cip1^ and *BAX* genes contain KLF4 response elements in the immediate vicinity of the documented p53 binding sites [40], [41]. They also contain additional putative response elements for both TFs, some of which are clustered. B: Whole genome identification of p53 and KLF4 response elements co-localised within 200 bp.

Both the *p21* ^WAF1/Cip1^ and the *BAX* promoters contain well documented REs for p53 [40], [41], and were shown to respond to and bind KLF4 [21], [22]. Here, we identified putative KLF4 REs in the vicinity of the p53 binding sites. We found a KLF4 RE immediately adjacent downstream to the p53 site in the *BAX* promoter, and one only 15 bp upstream in the *p21* promoter (Figure 5A). In addition, there were several other KLF4 as well as p53 REs in those regions. The whole genome analysis suggested that there is a significant scope for the KLF4-mediated regulation of the p53 response, as approximately 13% of putative p53 binding sites had a KLF4 site within 200 bp. In the case of approximately 2% of the REs of both TFs the REs overlapped and the binding would be mutually exclusive (Figure 5B).

## Discussion

### KLF4 Increases Target Specificity of p53

It has been shown *in vivo* that p53 and KLF4 synergistically activate the *p21* ^WAF1/Cip1^ promoter [23] and are bound simultaneously to the promoter regions of *p21* ^WAF1/Cip1^ and *BAX* [22]. Here, we present data which demonstrate the means by which the target selectivity of p53 may be provided: KLF4 increases through ternary complex formation the DNA-binding affinity of p53. However, the increase in affinity was observed only, if p53 bound DNA specifically via its DNA-binding domain. As p53 adopts different conformations, when bound specifically or non-specifically to DNA [17], we conclude that the KLF4-p53 interaction is far stronger for the ‘specific’ conformation that is achieved through binding of the DBD rather than the CTD. Taken together, our data suggest that increased levels of KLF4 modulate transcriptional activity of p53 by enriching it at promoters where both proteins can bind to DNA cooperatively. Importantly, the enhancement of the DNA binding was observed for both strong canonical p53 REs as well as for weak non-canonical REs. Given the abundance of non-canonical REs [4], [42], this will further expand the potential transcription regulation network of p53. We identified that a significant proportion of putative p53 REs are close to KLF4 REs. The scope of the p53-KLF4 interaction may, therefore, well extend beyond the *p21* ^WAF1/Cip1^ and *BAX* promoters. As the interaction between p53 and KLF4 only needs REs for both proteins within a certain distance range from each other, it is plausible that such loosely arranged, “fuzzy”, sites may readily appear and disappear in evolution, causing flexible re-wiring of transcription factor networks.

### Identification of Binding Sites in KLF4 and p53

**Figure 6:**
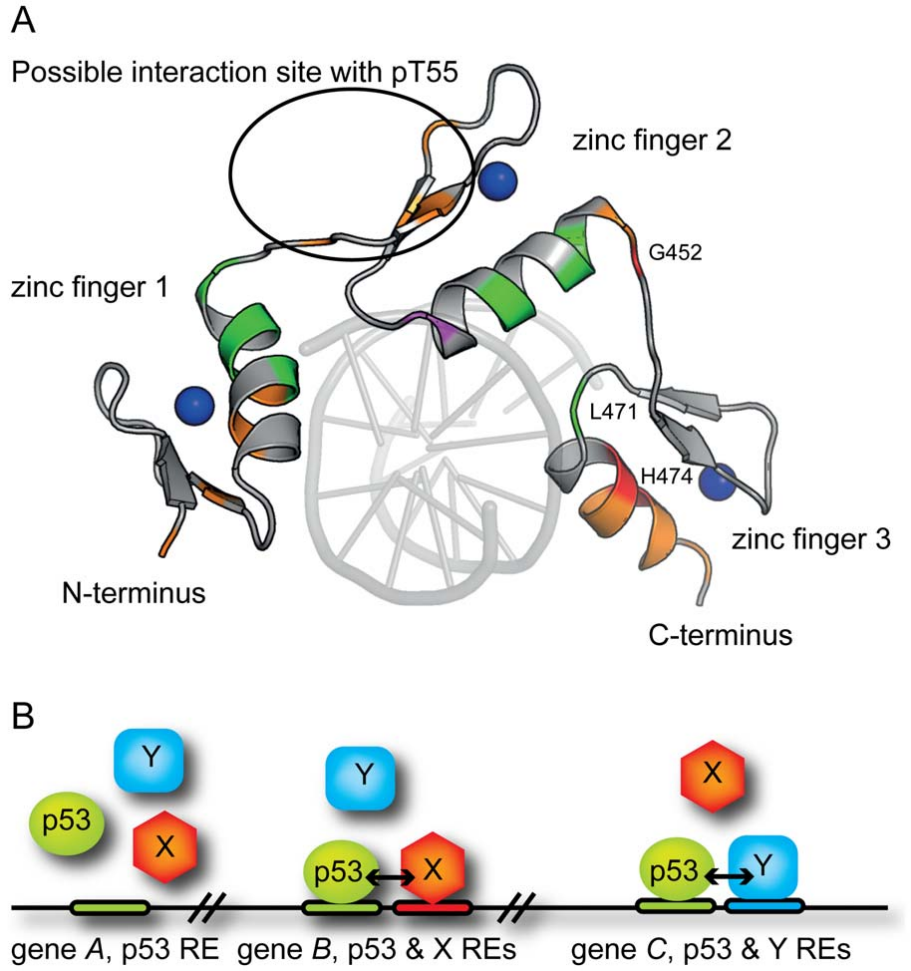
Interaction model for p53 and KLF4. A: View of the KLF4 zinc-finger domain bound to DNA (residues 395–479, human KLF4 sequence), PDB 2WBU [36]. Side-chains of residues involved in the interaction are shown (green- moderate (0.035–0.055), orange – intermediate (0.55–0.1) and red – large (>0.1) weighted chemical shift perturbations). Residues with signals shifted only in the presence of the phosphorylated peptide, cluster on the second zinc finger and are encircled. Zn-atoms are shown as blue spheres. The binding site observed between residues 386 and 395 is not shown as this part was absent in the crystal structure. Please note that DNA was not present in our NMR experiments. B: Cooperative binding of transcription factors to DNA increases their specificity. TFs are represented by shapes, corresponding REs by horizontal bars of the same colour, and genomic DNA by a black line. *Gene A*: DNA-binding specificity of p53 alone is not sufficient to guide it to a specific site. *Gene B*: Cooperative binding with another TF “X” (e.g., KLF4) mediated by protein-protein interactions would recruit both TFs to a specific locus containing both REs (p53/“X”). *Gene C*: Direct or indirect interactions with another TF “Y” would recruit both TFs to a p53/“Y” specific locus.

Co-IP assays had previously suggested an interaction between KLF4 and p53, believed to be localised to the p53NTD and the KLF4 zinc fingers regions [23]. Using NMR and fluorescence spectroscopy, we mapped interactions between the N-terminal domain of p53, p53NTD, and the zinc-finger domain of KLF4, KLF4 367–479. The overall binding affinity for this interaction was in the micromolar range in the absence of DNA. However, binding of KLF4 to DNA-bound full-length p53 was much stronger. This interaction was further enhanced by phosphorylation of p53NTD. KLF4 interacts with both the TAD1 and TAD2 regions of p53. These regions are also main interaction sites for proteins such as MDM2, p300, or BRCA2 [28], [43], [44]. p53 NTD – MDM2 interactions are characterised by a bleaching of resonances in the NMR spectra suggesting conformational exchange processes and binding in the lower micromolar range [35]. For the KLF4 binding to p53 NTD, we observed chemical shift perturbations but no bleaching of signals, indicating that the binding surface is well defined. The interaction site within KLF4 extended over the three zinc-fingers and a short unfolded region at their N-terminus (Figure 6A). The zinc fingers bind DNA [36] and are important for the activation of KLF4. Involved in this interaction are the p53 residues L22 and W23, as well as W53 and F54. Both L22/W23 and W53/F54 double mutants of p53 do not induce transcription of apoptosis and cell-cycle arrest genes [45], [46]. It is likely that, similar to p53-MDM2 and p53-p300 interactions, the p53-KLF4 interaction is also affected by these mutations, contributing to the loss of transactivation of cell-cycle arrest genes.

Overall, our data reveal the interaction sites of p53 and KLF4. Their disruption may be of medical importance in tumours which over-express KLF4, because KLF4 has been shown to switch the p53 response from apoptosis towards cell-cycle arrest in cancer cells after γ–irradiation [22].

### Phosphorylation of p53 Increases its Affinity for KLF4

Both TAD1 and TAD2 of p53 are extensively phosphorylated on multiple residues in response to carcinogenic stress [47]. Phosphorylation of p53 at S15, S20, and S46 drives the p53 response towards apoptosis, and phosphorylation of S46 and T55 promotes p53-dependent transcription of cell-cycle arrest genes [48], [49], [50], [51], [52], [53]. Our data (Table 1, Figure S6) show that phosphorylation of S46 or T55 increased the affinity of the p53NTD to KLF4 significantly (8 fold), while modifications of other serine or threonine residues (15, 18, 20, 33 and 37) had smaller effects (3–4 fold). Furthermore, multi-site phosphorylation on all available serine and threonine residues in p53 (10–57) further enhanced the interaction with KLF4 three-fold with respect to the most potent single site phosphorylation on S46/T55, indicating cumulative effects. A similar, though more pronounced tendency, is observed with p300 domains, [28], [54] suggesting that target protein binding affinity enhancements in response to multi-site p53 phosphorylation may be a general mechanism for enhancing the p53 transcriptional response. Taken together, phosphorylation(s) of p53, in particular those associated with the cell-cycle arrest response, influence the p53-KLF4 interaction.

In the absence of phosphorylation the observed effect of KLF4 on p53 DNA-binding affinity (3–4 fold increase) is not large enough to explain transcriptional selectivity based purely on this interaction. However, upon phosphorylation of p53 at S46 and T55 the effect of KLF4 on p53 DNA-binding affinity should increase proportionally as these equilibria are linked (Figure S8).

### Conclusions

Given the involvement of p53 in a variety of signalling events, a simple on/off-switch mechanism is unlikely to provide the p53 network with enough flexibility to transcribe many different genes selectively. Several different signals such as p53 activation, post-translational modifications or histone modifications have to be integrated to guarantee transcriptional selectivity (reviewed in [2], [10]). In principle, the cooperative binding of two transcription factors, such as p53 and KLF4, may contribute to the selection of only a subset of all available cognate sites. Here, we suggest a model where KLF4 guides p53 towards certain promoters, provided that both KLF4 and p53 response elements are present within a certain distance (Figure 6B). There, since p53 is a tetramer and has four N-termini available, it may simultaneously interact with KLF4 and possibly other, yet to be identified, transcription factors, p300/CBP [35], and components of the transcriptional machinery such as TFIIH [55]. Thereby p53 would combine or “bridge” several regulatory interactions occurring on DNA, and ensure transcriptional activation that is specific to the KLF4/p53 combination.

## Supporting Information

Figure S1 ***In vitro*** **characterisation of KLF4.**
(TIF)

Figure S2 **2D NMR experiments with labelled p53DBD/p53TC and KLF4 (271–479).**
(TIF)

Figure S3 **Chemical shift perturbation map for the interaction between labelled KLF4 (367–479) and (phosphorylated) N-terminal p53.**
(TIF)

Figure S4 **2D NMR experiments with labelled KLF4 (367–479) and p53TC.**
(TIF)

Figure S5 **HSQC of labelled KLF4 (271–390).**
(TIF)

Figure S6 **Fluorescence anisotropy titrations with N-terminal peptides of p53 and KLF4.**
(TIF)

Figure S7 **Cooperative fluorescence anisotropy titrations using p53, DNA encoding weak p53REs and KLF4.**
(TIF)

Figure S8 **Scheme of the thermodynamic cycle for phosphorylation-mediated binding of KLF4 to p53.**
(TIF)

Table S1 **Fluorescently labelled DNA.**
(PDF)

Table S2 **DNA-binding affinities of KLF4 determined by fluorescence anisotropy titrations.**
(PDF)

Table S3 **Chemical shifts for KLF4 367–479.**
(PDF)

Table S4 **Cooperative binding assay of p53 and CP2/HSF1/YY1.**
(PDF)

Table S5 **Cooperative binding of KLF4 and p53 to DNA using FA210 buffer.**
(PDF)

Text S1 **Supplementary materials and methods section.**
(DOC)

Text S2 **Supplementary results section on the** ***in vitro*** **characterisation of KLF4.**
(DOC)
