# Supplementary figures and images for: Molecular Basis for Modulation of the p53 Target Selectivity by KLF4
Source: PLoS One. 2012 Oct 30;7(10):e48252. doi: 10.1371/journal.pone.0048252 (PMC3484126; doi:10.1371/journal.pone.0048252)

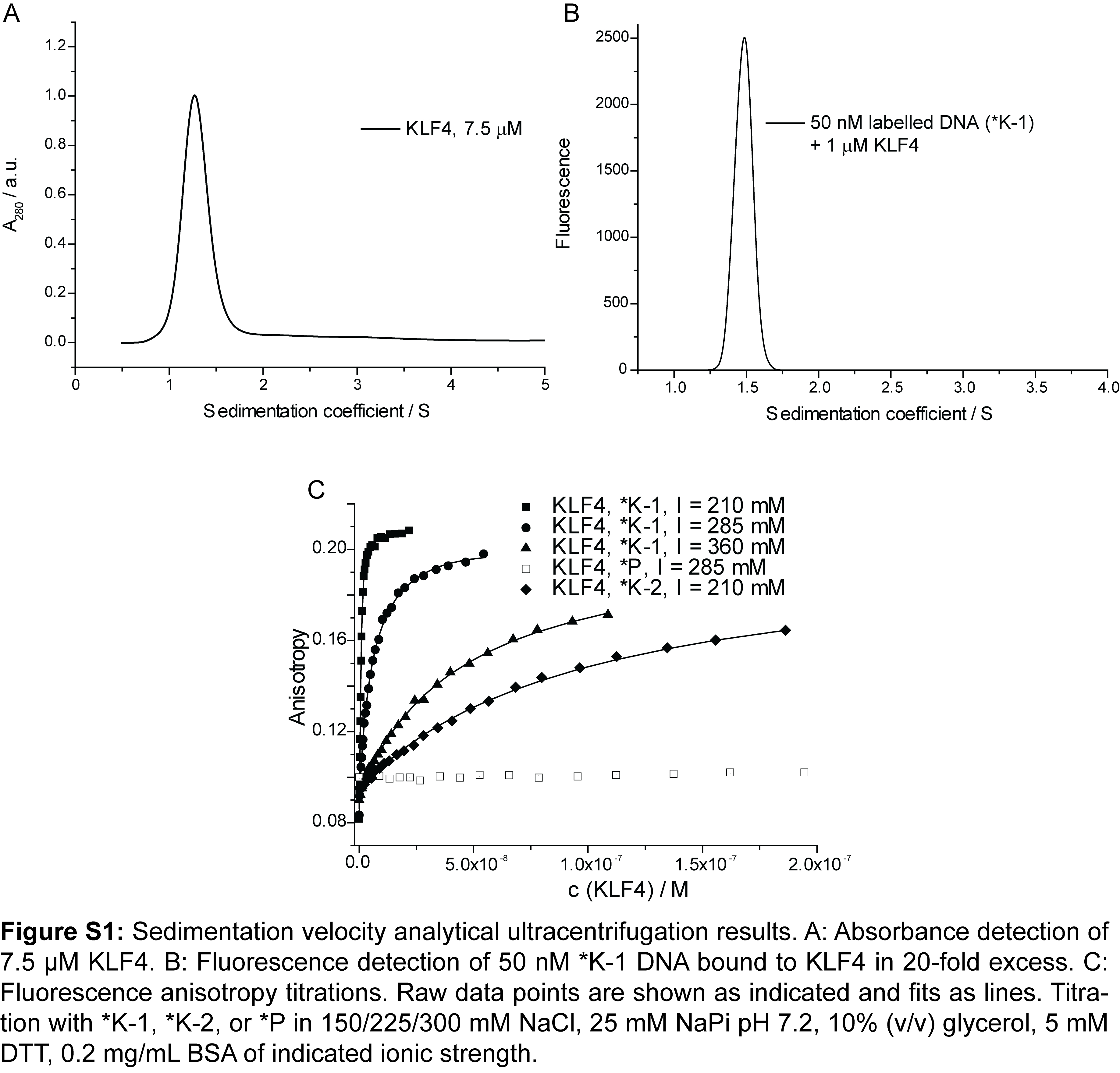

Supplement: Figure S1 — In vitro characterisation of KLF4. (TIF) [file pone.0048252.s001.tif]

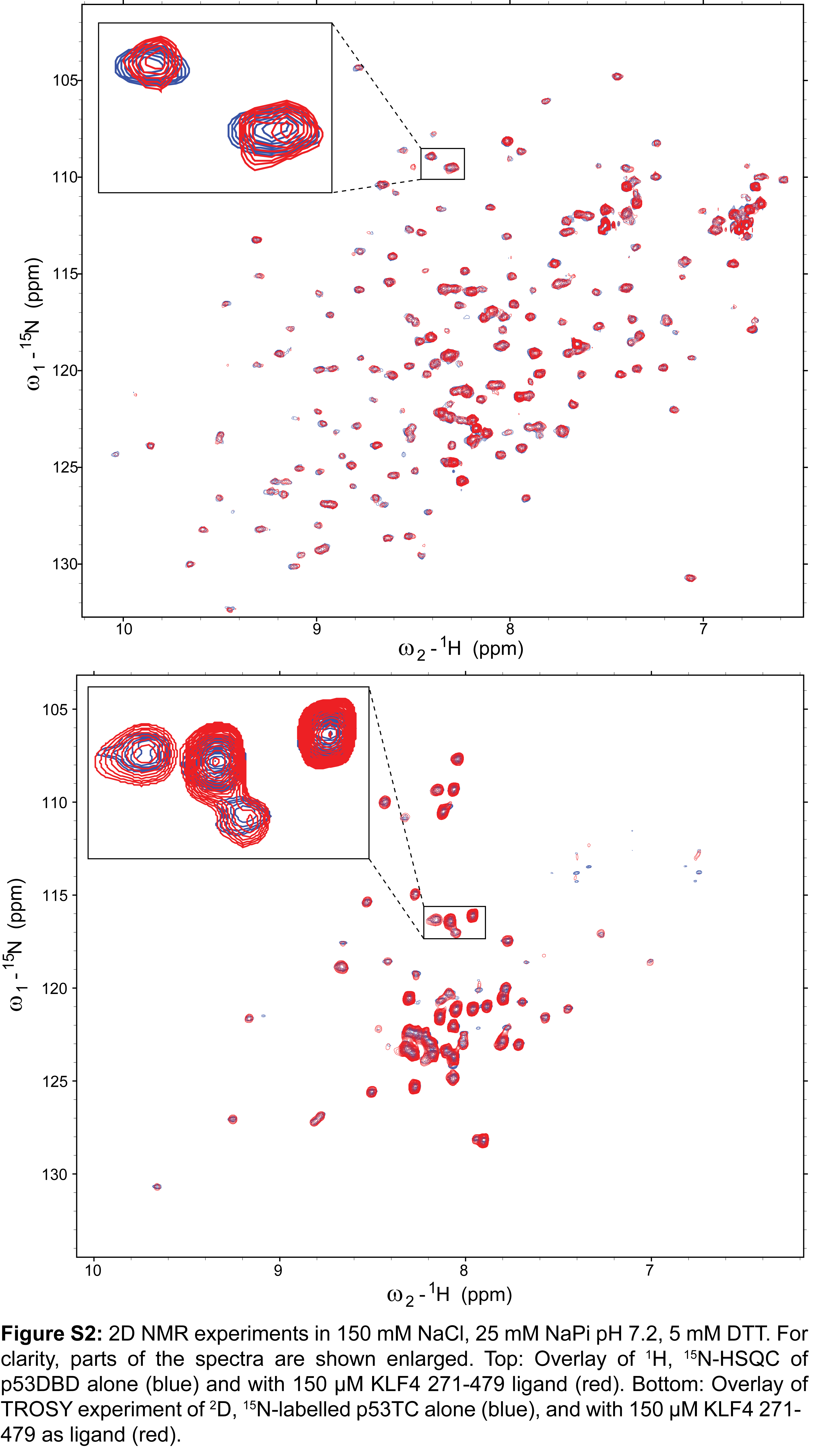

Supplement: Figure S2 — 2D NMR experiments with labelled p53DBD/p53TC and KLF4 (271–479). (TIF) [file pone.0048252.s002.tif]

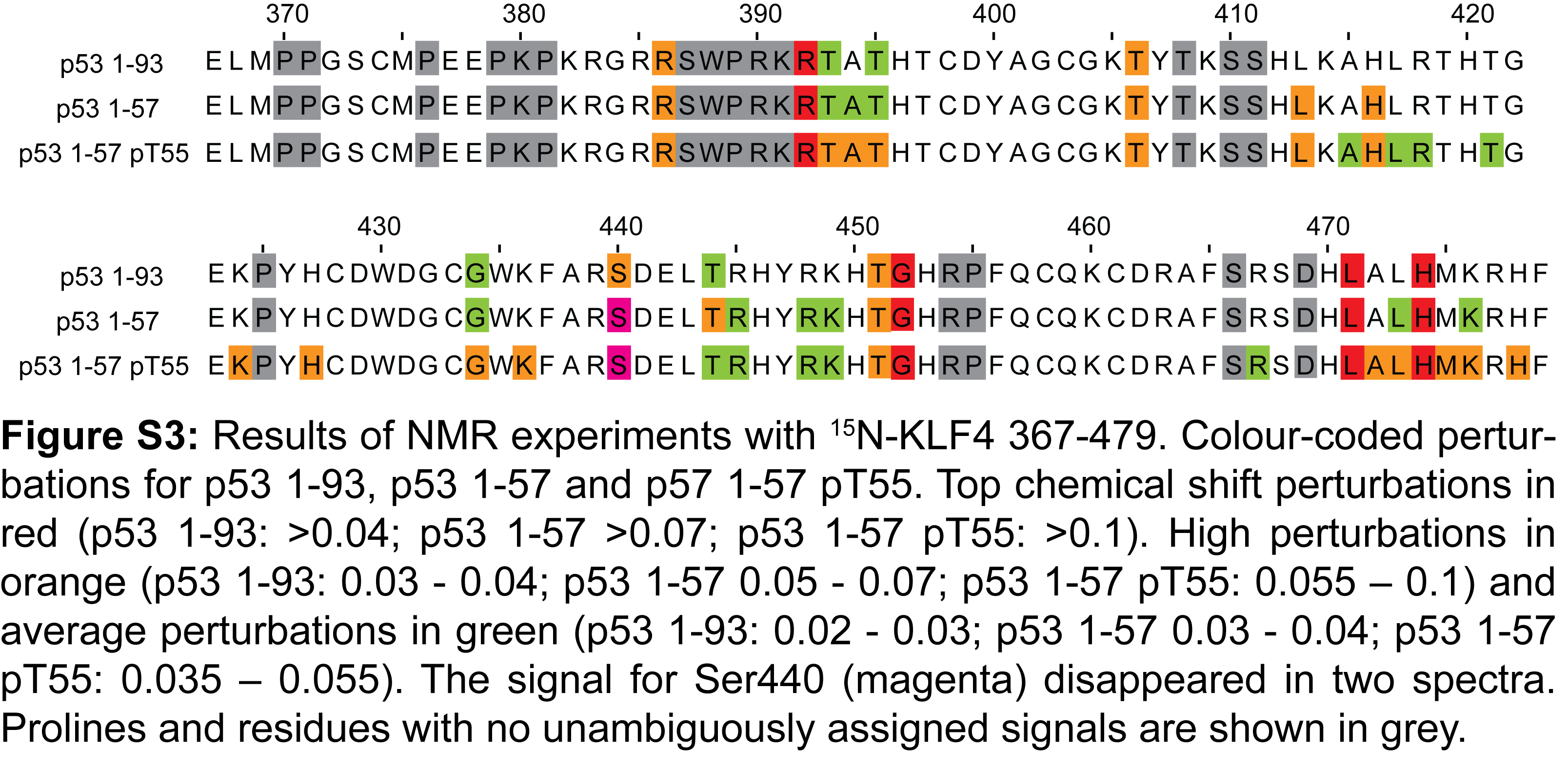

Supplement: Figure S3 — Chemical shift perturbation map for the interaction between labelled KLF4 (367–479) and (phosphorylated) N-terminal p53. (TIF) [file pone.0048252.s003.tif]

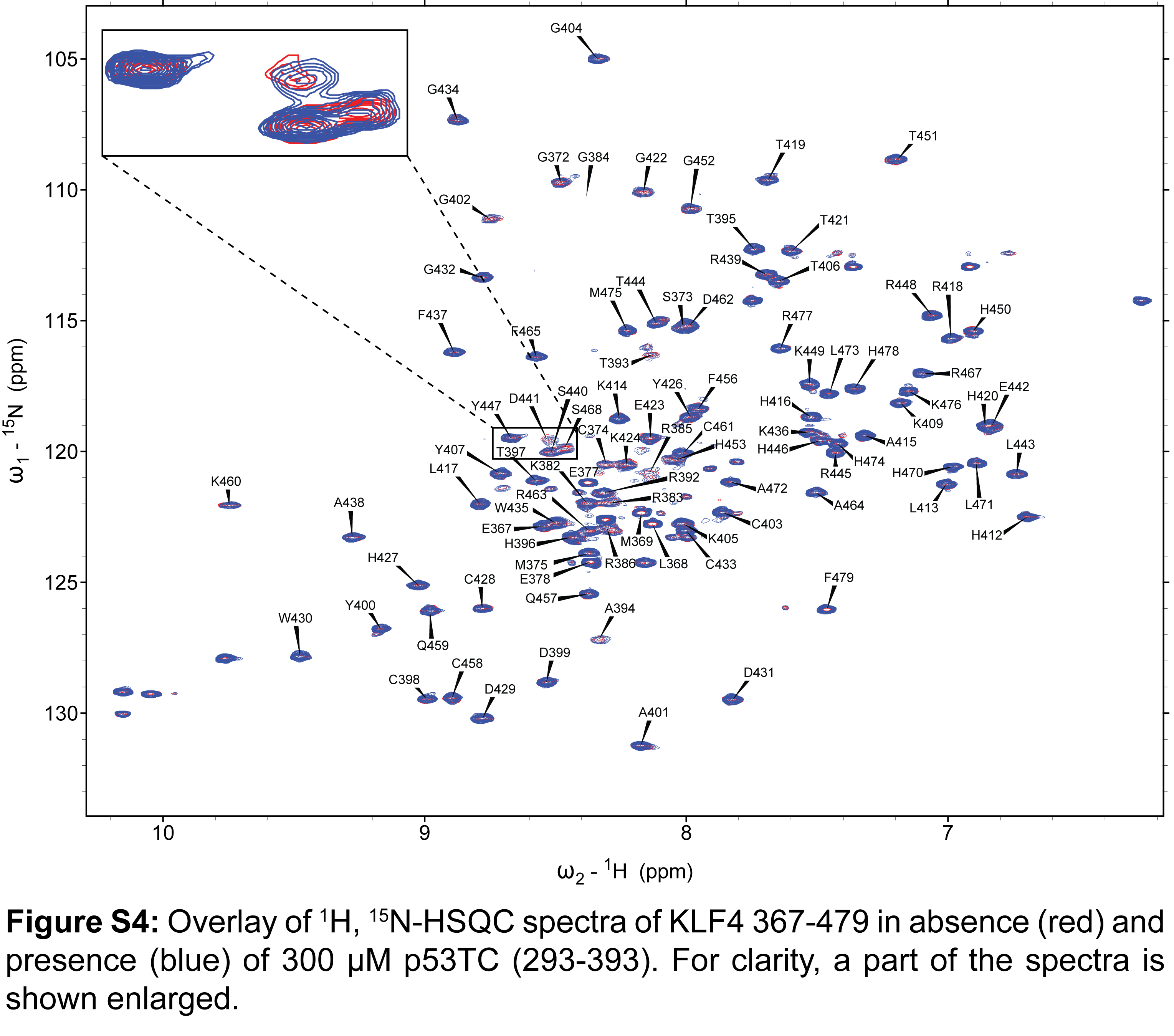

Supplement: Figure S4 — 2D NMR experiments with labelled KLF4 (367–479) and p53TC. (TIF) [file pone.0048252.s004.tif]

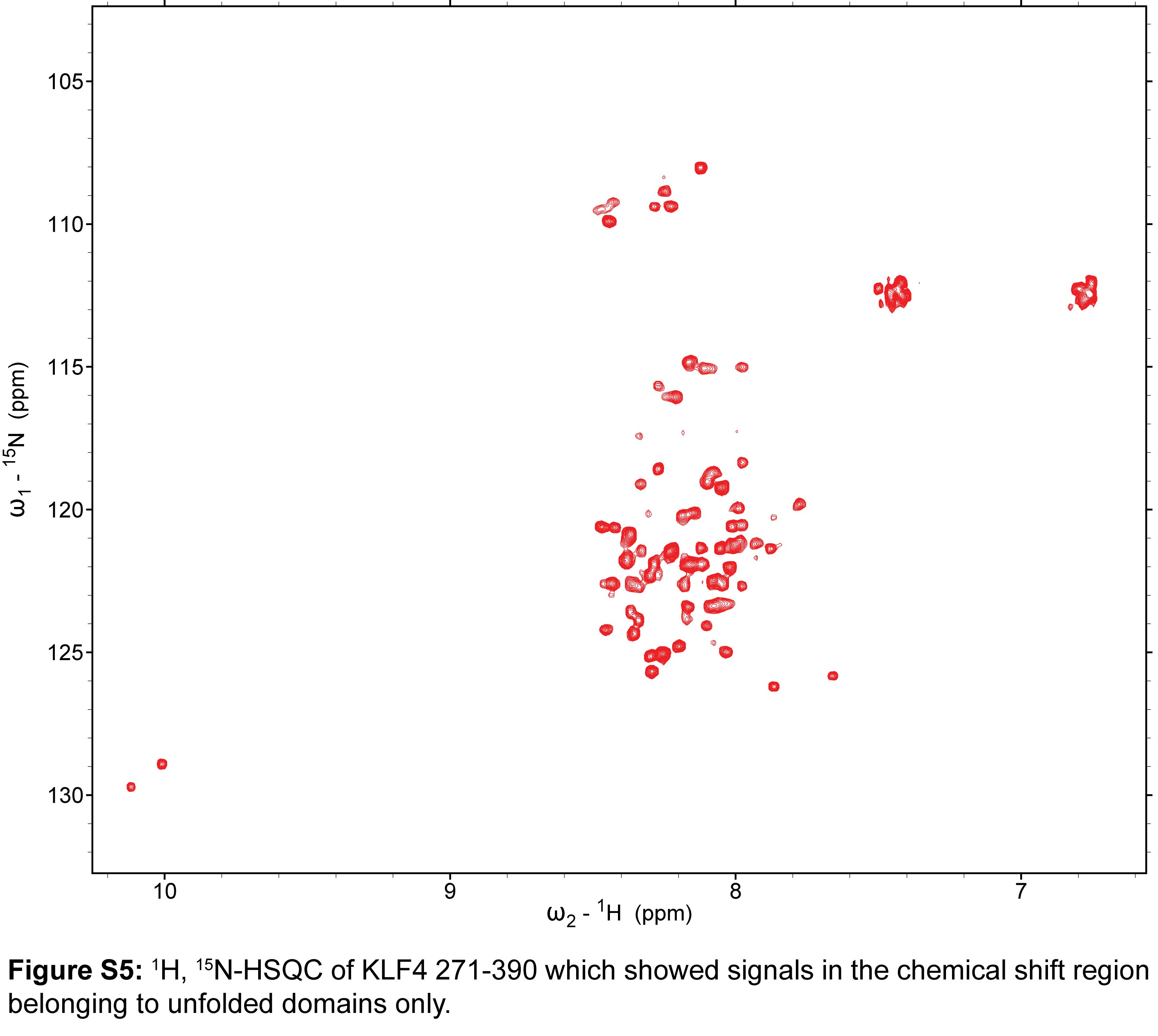

Supplement: Figure S5 — HSQC of labelled KLF4 (271–390). (TIF) [file pone.0048252.s005.tif]

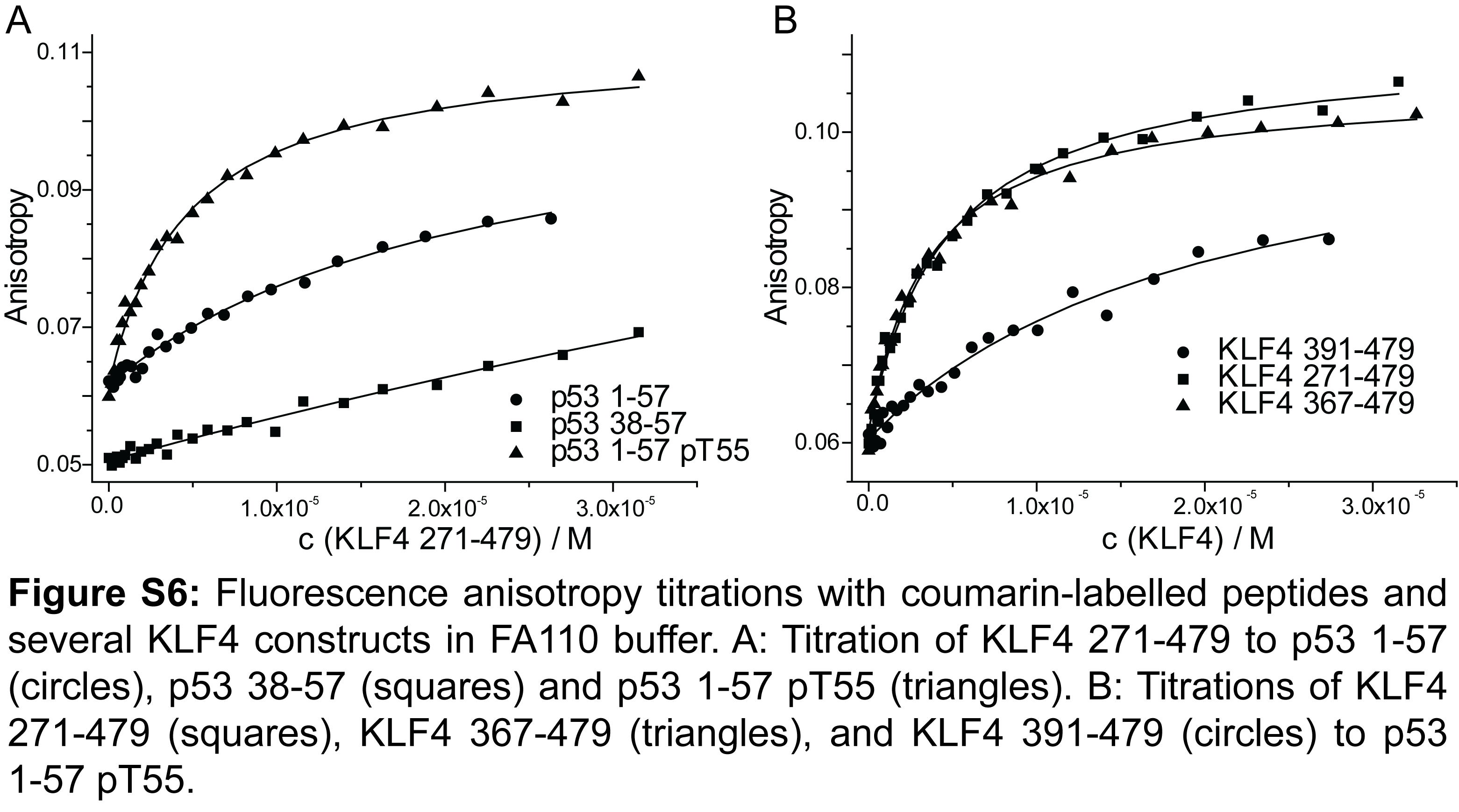

Supplement: Figure S6 — Fluorescence anisotropy titrations with N-terminal peptides of p53 and KLF4. (TIF) [file pone.0048252.s006.tif]

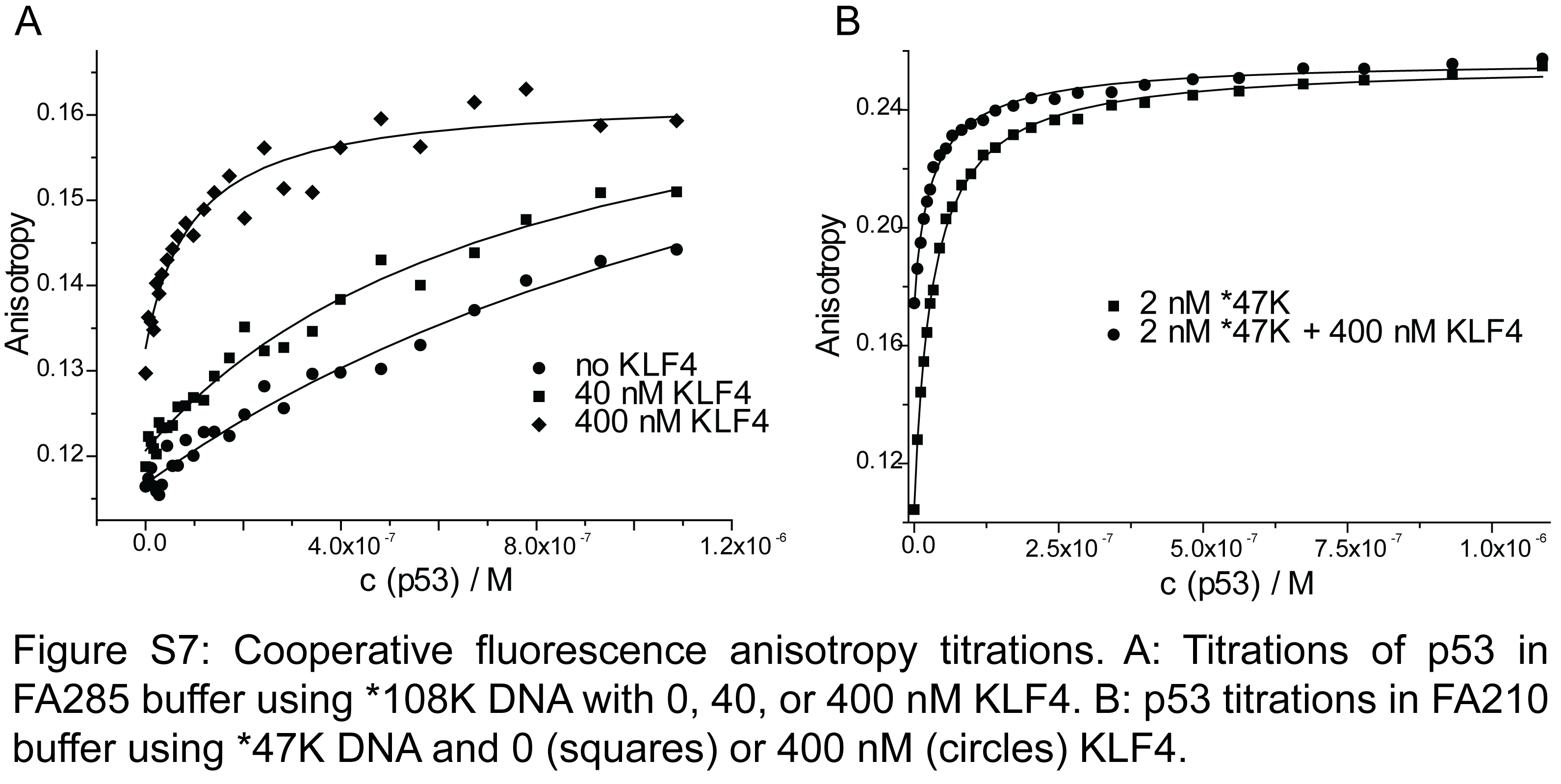

Supplement: Figure S7 — Cooperative fluorescence anisotropy titrations using p53, DNA encoding weak p53REs and KLF4. (TIF) [file pone.0048252.s007.tif]

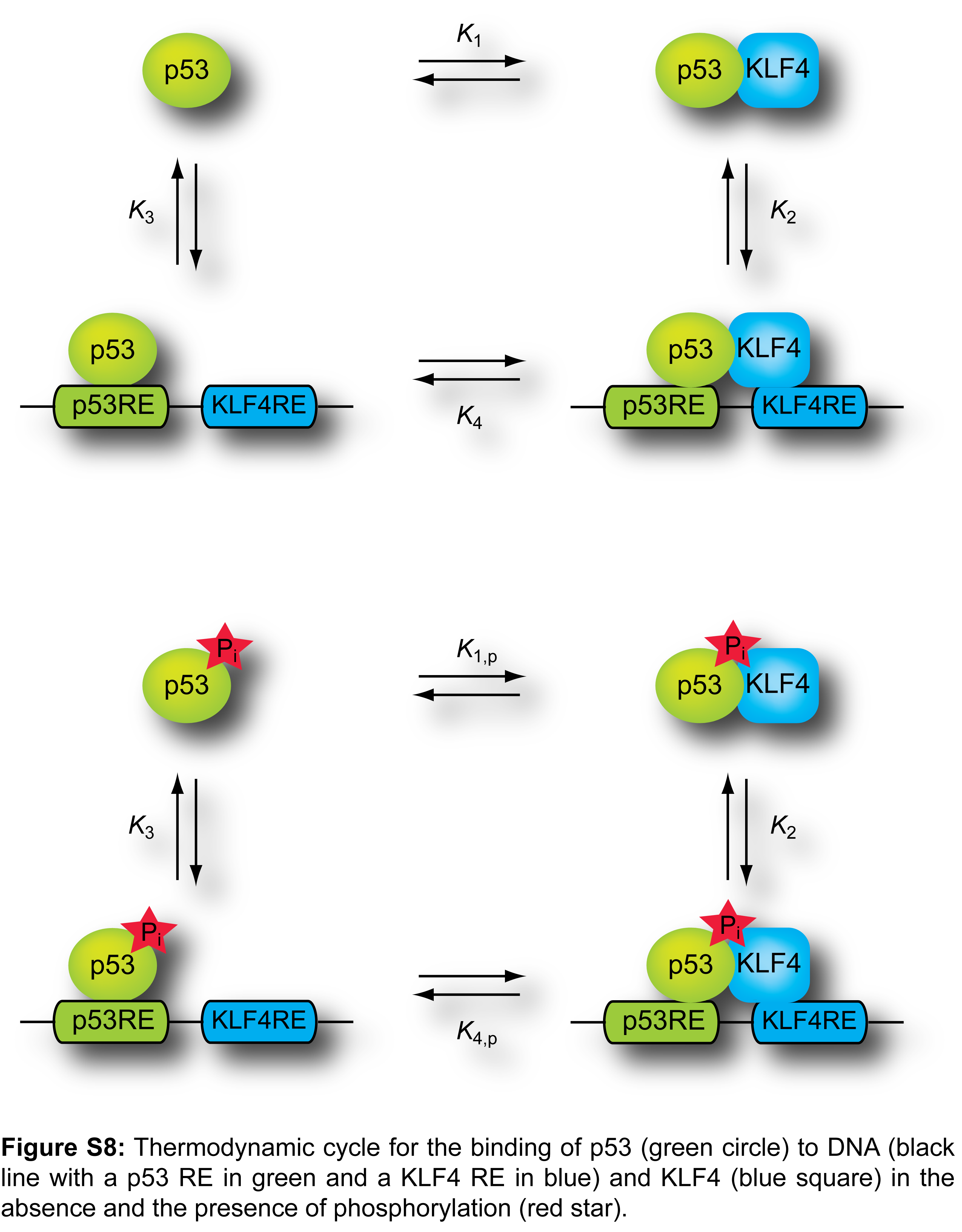

Supplement: Figure S8 — Scheme of the thermodynamic cycle for phosphorylation-mediated binding of KLF4 to p53. (TIF) [file pone.0048252.s008.tif]
