# Supplementary material for: Molecular Basis for Modulation of the p53 Target Selectivity by KLF4
Source: PLoS One. 2012 Oct 30;7(10):e48252. doi: 10.1371/journal.pone.0048252 (PMC3484126; doi:10.1371/journal.pone.0048252)
Supplement: Table S1 — Fluorescently labelled DNA. (PDF) [file pone.0048252.s009.pdf]

Table S1: Fluorescently labelled DNA. *denotes the label (X = fluorescein, Y = Alexa488), *P* if a p53RE (bold letters) is present, *n* the spacer length, and *K* if a Klf4RE (italic letters) is present. Non-canonical three quarter-sites are highlighted in green for the constructs *108K and *47K. The affinity-defining bases C4, G7, C14 and G17 are underlined and changes to the canonical bases indicated in red.

*P	Y**GGACATGTCCGGACATGTCC**
*PK	Y**GGACATGTCCGGACATGTCC***AGGGTGTGGCCCGC*
*P5K	Y**GGACATGTCCGGACATGTCC**AGCTC*AGGGTGTGGCCCGC*
*P10K	Y**GGACATGTCCGGACATGTCC**AGCTCTGGC*AAGGGTGTGGCCCGC*
*P30K	Y**GGACATGTCCGGACATGTCC**AGCTCTGGCATAGAAGAGGCTGGTGGCTAT*AGGGTGTGGCCCGC*
*P88K	Y**GGACATGTCCGGACATGTCC**AGCTCTGGCATAGAAGAGGCTGGTGGCTATTTTGTCCTTGGGCTGCCTG<br>TTTTCAGGTGAGGAAGGGGATGGTAGGAGACAGGAGACC*AGGGTGTGGCCCGC*
*P185K	Y**GGACATGTCCGGACATGTCC**AGCTCTGGCATAGAAGAGGCTGGTGGCTATTTTGTCCTTGGGCTGCCTG<br>TTTTCAGGTGAGGAAGGGGATGGTAGGAGACAGGAGACCTCTAAAGACCCCAGGTAAACCTTAGCCTGT<br>TACTCTGAACAGGGTATGTGATCTGCCAGCAGATCCTTGCGACAGGG*AGGGTGTGGCCCGC*
*P1000K	Y**GGACATGTCCGGACATGTCC**AGCTCTGGCATAGAAGAGGCTGGTGGCTATTTTGTCCTTGGGCTGCCTG<br>TTTTCAGGTGAGGAAGGGGATGGTAGGAGACAGGAGACCTCTAAAGACCCCAGGTAAACCTTAGCCTGT<br>TACTCTGAACAGGGTATGTGATCTGCCAGCAGATCCTTGCGACAGGGCTGGGATCTGATGCATGCGTGCT<br>TGTGTGAGTGTGTGCTGGGAGTCAGATTCTGTGTGTGACTTTTAACAGCCTGCTCCCTTGCCTTCTTCAGG<br>GCAGAAGTCCTCCCTTAGAGTGTGTCTGGGAACACATTCAAGTGCATGGTTGCAAACTTTTTTTTTTAAAG<br>CACTGAATAGTACTAGACACTTAGTAGGTACTTAAGAAATATTGAATGTCGTGGTGGTGGTGAGCAAGGG<br>TGGGCGCGCCGACCCAGCTTTCTTGTACAAAGTTGGCATTATAAGAAAGCATTGCTTATCAATTTGTTGCA<br>ACGAACAGGTCACTATCAGTCAAAATAAAATCATTATTTGCCATCCAGCTGATATCCCCTATAGTGAGTCG<br>TATTACATGGTCATAGCTGTTTCCTGCATATGAATTCGAAGCTTGATCCGGCTGCTAACAAAGCCCGAAAG<br>GAAGCTGAGTTGGCTGCTGCCACCGCTGAGCAATAACTAGCATAACCCCTTGGGGCCTCTAAACGGGTCT<br>TGAGGGGTTTTTTGCTGAAAGGAGGAACTATATCCGGATCTGGCGTAATAGCGAAGAGGCCCGCACCGA<br>TCGCCCTTCCCAACAGTTGCGCAGCCTGAATGGCGAATGGGACGCGCCCTGTAGCGGCGCATTAAGCGCG<br>GCGGGTGTGGTGGTTACGCGCAGCGTGACCGCTACACTTGCCAGCGCCCTAGCGCCCGCTCCTTTCGCTTT<br>CTTCCCTTCCTTTCTCGCCACGTTCGCCGGCTTTCCCCGTCAAGCTCTAAATCGGGGGCTCCCTTTAGGGTT<br>CCGATTTAGAGCTTTACGGCACCTCGACCGCAAAA*AGGGTGTGGCCCGC*
*K-1	X*AGGGTGTGGCCCGC*
*47K	XATCCCAGGTCACCCTGACCTAGCTCTGGCATAGAAGAGGCTGGTGGCTAT*AGGGTGTGGCCCGC*
*K-2	X*GGGCCACCCCGCC*
*108K	XGAAGATCTCCCAAGATCTTGAGCTCTGGCATAGAAGAGGCTGGTGGCTATTTTGTCCTTGGGCTGCCTGT<br>TTTCAGGTGAGGAAGGGGATGGTAGGAGACAGGAGACC*AGGGTGTGGCCCGC*
