# Supplementary material for: Molecular Basis for Modulation of the p53 Target Selectivity by KLF4
Source: PLoS One. 2012 Oct 30;7(10):e48252. doi: 10.1371/journal.pone.0048252 (PMC3484126; doi:10.1371/journal.pone.0048252)
Supplement: Table S2 — DNA-binding affinities of KLF4 determined by fluorescence anisotropy titrations. (PDF) [file pone.0048252.s010.pdf]

Table S2: DNA-binding affinities of KLF4 determined by fluorescence anisotropy titrations.

DNA	KLF4 construct	Ionic strength / mM	K_d ± SD / nM
*K-1	FL	210	0.7 ± 0.2
	FL	285	4 ± 0.6
	FL	360	44 ± 4
	179-479	285	3 ± 1
	271-479	285	2 ± 0.4
	391-479	285	2 ± 0.4
*K-2	FL	210	85 ± 14
*P	FL	210	>1000
