# Supplementary material for: Molecular Basis for Modulation of the p53 Target Selectivity by KLF4
Source: PLoS One. 2012 Oct 30;7(10):e48252. doi: 10.1371/journal.pone.0048252 (PMC3484126; doi:10.1371/journal.pone.0048252)
Supplement: Table S3 — Chemical shifts for KLF4 367–479. (PDF) [file pone.0048252.s011.pdf]

Table S3: Chemical shifts for KLF4 367-479 in NMR buffer. Signals for residues K380, S387, W388, R390, K391, T408, S410, S411, R454, S466, and D469 were either missing or could not unambiguously assigned. Eight proline residues were not visible with the pulse sequences used (P370, 371, 376, 379, 381, 389, 425, and 455).

Residue	C_{α}	C_{α}p	C_{β}	C_{β}p	C(O)	C(O)p	H(N)	N
E367	54.18	55.92	27.3	61.11	173.5	171.9	8.545	122.9
L368	52.45	54.14	39.62	27.31	174.3	173.5	8.127	122.8
M369	50.31	52.43	29.8	39.61	170.9	174.3	8.168	122.4
G372	42.61	60.76	-	29.3	171.7	174.9	8.484	109.8
S373	56.05	42.63	61.42	-	171.5	171.7	8.01	115.2
C374	55.69	55.93	25.55	61.4	171.5	171.5	8.309	120.5
M375	50.7	55.61	29.47	25.57	172.2	171.4	8.372	123.9
E377	53.48	60.46	27.94	29.37	173.5	173.9	8.377	121.2
E378	51.62	53.45	27.25	27.96	171.7	173.5	8.36	124.2
K382	53.73	60.42	30.32	29.44	174	174.2	8.379	122
R383	53.46	53.71	28.23	30.34	174	174	8.296	122
G384	42.5	53.5	-	28.14	171.2	174	8.391	110.3
R385	53.49	42.44	28.15	-	173.7	171.2	8.139	120.8
R386	51.44	53.58	30.56	28.21	-	173.7	8.3	122.9
R392	53.42	60.54	28.2	29.35	173.6	174	8.318	121.6
T393	59.12	53.44	67.25	28.2	171.2	173.6	8.138	116.3
A394	49.81	59.14	16.7	67.22	174.1	171.2	8.332	127.2
T395	58.66	49.66	67.69	16.79	170.8	174.1	7.74	112.3
H396	52.9	58.66	28.68	67.76	172	170.8	8.429	123.3
T397	59.22	52.78	68.06	28.69	170.4	171.9	8.577	121.1
C398	59.12	59.13	27.69	68.08	173.1	170.4	8.982	129.4
D399	51.27	59.14	38.29	27.64	173.4	173.1	8.532	128.8
Y400	57.71	51.26	36.12	38.24	174.1	173.4	9.161	126.7
A401	51.28	57.7	15.13	36.05	176	174.1	8.187	131.2
G402	43.27	51.25	-	15.21	171	176	8.742	111.1
C403	58.41	43.1	26.87	-	173.5	171	7.851	122.3
G404	43.26	58.37	-	26.9	171.8	173.5	8.333	105.1
K405	55.49	43.24	30.39	-	172.4	171.8	8	122.8
T406	57.78	55.51	68.91	30.39	170	172.4	7.639	113.5
Y407	54.91	57.79	41.14	68.88	173	170	8.71	120.8
K409	51.84	60.22	32.76	66.84	174	172.1	7.18	118.2
H412	54.25	58.78	28.81	58.8	175.5	174.9	6.697	122.5
L413	55.18	54.27	37.61	28.81	174.9	175.5	7.007	121.3
K414	57.59	55.09	29.44	37.61	176.3	174.8	8.254	118.8
A415	52.25	57.57	15.32	29.44	177.4	176.3	7.312	119.4
H416	56.36	52.23	25.36	15.29	175.8	177.4	7.52	118.7
L417	55.94	56.45	39.1	25.29	176.5	175.8	8.785	122
R418	55.68	55.88	27.33	39.09	175.5	176.5	6.995	115.7
T419	61.08	55.67	66.43	27.35	172.7	175.5	7.694	109.7
H420	52.45	61.02	25.16	66.48	172.7	172.7	6.87	119
T421	59.14	52.38	67.45	25.18	172.1	172.7	7.599	112.3
G422	42.35	59.04	-	67.43	171.2	172.1	8.156	110.1
E423	54.76	42.35	27.71	-	173.9	171.2	8.121	119.6
K424	51.26	54.71	31.11	27.73	171.1	173.9	8.241	120.6
Y426	55.09	60.91	35.37	29.66	171.9	173.6	7.97	118.6
H427	51.81	55.16	29.7	35.39	171.6	171.9	9.012	125.1
C428	59.28	51.76	27.56	29.6	174	171.6	8.773	126
D429	51.14	59.22	38.4	27.48	173.8	174	8.767	130.2
W430	56.37	51.19	27.12	38.32	174.4	173.8	9.472	127.9
D431	53.95	56.37	38.03	27.11	174.4	174.4	7.81	129.5
G432	43.2	53.88	-	37.94	171.3	174.4	8.776	113.4
C433	58.8	43.1	27.57	-	173.7	171.3	8.003	123
G434	43.17	58.71	-	27.63	172.4	173.7	8.869	107.5
W435	57.55	43.2	26.56	-	172.4	172.4	8.494	122.7
K436	52.13	57.57	33.32	26.59	171.8	172.4	7.531	119.3
F437	54.74	52.15	41.37	33.24	172	171.7	8.88	116.2
A438	51.55	54.76	17.66	41.31	174.8	172	9.27	123.3
R439	51.26	51.55	31.88	17.72	174.2	174.8	7.691	113.2
S440	58.49	51.27	59.3	31.81	174.2	174.3	8.555	120.2
D441	53.7	58.4	36.9	59.39	175.7	174.3	8.532	119.6
E442	55.7	53.7	27.5	36.93	176.3	175.7	6.833	119
L443	55.17	55.6	37.21	27.35	174.7	176.3	6.732	120.9
T444	64.05	55.13	65.52	37.22	174.1	174.8	8.1	115
R445	57.1	64.17	27.66	65.5	176	174.1	7.43	120.1
H446	56.41	57.07	26.08	27.62	173.7	176	7.488	119.6
Y447	58.93	56.35	35.97	26.13	174.7	173.7	8.657	119.5
R448	55.23	58.93	27.38	35.93	175.4	174.7	7.059	114.8
K449	55.08	55.13	28.66	27.35	175.2	175.4	7.526	117.4
H450	52.9	55.07	24.05	28.55	172.8	175.2	6.899	115.4
T451	58.95	52.9	66.8	24.07	171.7	172.8	7.183	108.8
G452	42.53	58.79	-	66.71	171	171.7	7.986	110.8
H453	53.82	42.51	28.48	-	172.6	171	8.066	120.4
F456	54.73	60.87	35.6	29.48	171.8	173.8	7.946	118.4
Q457	52.21	54.71	28.27	35.61	172.2	171.8	8.371	125.4
C458	58.45	52.22	26.71	28.28	173.7	172.2	8.882	129.4
Q459	54.22	58.46	26.73	26.73	174	173.7	8.981	126.1
K460	53.71	54.24	30.46	26.73	173	174	9.739	122.1
C461	55.94	53.57	27.8	30.44	170.3	173	8.005	120.1
D462	52.41	55.85	37.95	27.79	174.1	170.4	7.986	115.2
R463	54.54	52.33	28.46	37.97	170.8	174.1	8.367	123
A464	47.7	54.51	19.77	28.41	173.2	170.8	7.501	121.6
F465	54.69	47.63	41.4	19.8	172.8	173.2	8.571	116.3
R467	51.41	57.62	32.09	61.82	173.8	171.1	7.082	117
S468	-	51.4	59.13	32.05	-	173.8	8.461	119.9
H470	54.51	53.41	28.85	35.61	175.3	176.1	6.971	120.5
L471	54.94	54.48	37.52	28.83	174.5	175.3	6.887	120.4
A472	52.85	54.98	15.02	37.46	178	174.5	7.824	121.1
L473	54.97	52.83	39.25	15	176.8	178	7.447	117.8
H474	56.42	54.97	25.95	39.23	174.1	176.8	7.414	119.7
M475	54.4	56.4	27.68	25.89	175.8	174.1	8.221	115.4
K476	55.86	54.42	29.34	27.59	175.2	175.8	7.144	117.7
R477	53.54	55.85	26.6	29.35	173.9	175.2	7.639	116.1
H478	52.9	53.54	26.54	26.48	171.1	173.9	7.35	117.6
F479	56.36	52.9	37.33	26.48	177.8	171.1	7.458	126
