# Supplementary material for: Molecular Basis for Modulation of the p53 Target Selectivity by KLF4
Source: PLoS One. 2012 Oct 30;7(10):e48252. doi: 10.1371/journal.pone.0048252 (PMC3484126; doi:10.1371/journal.pone.0048252)
Supplement: Table S4 — Cooperative binding assay of p53 and CP2/HSF1/YY1. (PDF) [file pone.0048252.s012.pdf]

Table S4: Results for cooperative binding assay. p53-DNA binding affinities measured by fluorescence anisotropy titrations. FA buffer of indicated ionic strength was used.

DNA	Ionic strength / mM	Protein	c / nM	K_d / nM
*P88C	160	CP2	0	6.9
			400	15
			1500	5.6
*P88H	210	HSF1	0	5.1
			400	15
			1500	32
			5000	21
*P88Y	210	YY1	0	4.2
			1500	4.2
			5000	4.5
