# Supplementary material for: Molecular Basis for Modulation of the p53 Target Selectivity by KLF4
Source: PLoS One. 2012 Oct 30;7(10):e48252. doi: 10.1371/journal.pone.0048252 (PMC3484126; doi:10.1371/journal.pone.0048252)
Supplement: Table S5 — Cooperative binding of KLF4 and p53 to DNA using FA210 buffer. (PDF) [file pone.0048252.s013.pdf]

Table S5: Results of fluorescence anisotropy titrations for DNA binding affinities of p53 in dependence of added KLF4 and spacing base pairs. FA 210 buffer was used.

DNA	*c* (KLF4) / nM	$K_d \pm$ SD / nM
*PK	0	3.7 ± 0.4
	400	2.8 ± 0.3
*P5K	0	4.6 ± 0.6
	400	2.5 ± 0.9
*P10K	0	5.1 ± 1.3
	400	2.4 ± 0.6
*P30K	0	11 ± 1.6
	400	2.9 ± 0.0
