# Supplementary material for: Molecular Basis for Modulation of the p53 Target Selectivity by KLF4
Source: PLoS One. 2012 Oct 30;7(10):e48252. doi: 10.1371/journal.pone.0048252 (PMC3484126; doi:10.1371/journal.pone.0048252)
Supplement: Text S1 — Supplementary materials and methods section. (DOC) [file pone.0048252.s014.doc]

# Supplementary materials and methods

## Gene cloning and protein preparation

For full-length and truncated versions of p53 we used a super-stable mutant, which has four mutations in the core domain (QM-Hsp53): M133L/V203A/N239Y/N268D . This mutant stabilises the protein without changing the structure and DNA-binding abilities of p53 *in vitro* , and is ideal for biophysical studies. KLF4 (isoform 2) was amplified from a clone provided by the MGC collection (distributed via Geneservice). All inserts were cloned into a pET24a-HLTEV plasmid containing an N-terminal 6xHis purification tag, a lipoyl domain for improved solubility and a TEV-protease cleavage site. Additionally, a KLF4 construct containing a C-terminal FlAsH tag was prepared.

Expression and purification of p53 family constructs was carried as described earlier . KLF4 constructs were overexpressed in 2xTy medium using *E. coli* BL21 or B834 cells (Novagen) at 18-20 °C for 16-20 h. Labelled protein for NMR was grown in M9 minimal medium supplemented with a vitamin mix together with 1 g/L ^15^NH_4_Cl and ^13^C *D*-glucose. For deuterated samples M9 medium was prepared using D_2_O. Proteins were purified using standard Ni-affinity chromatography protocols. Subsequently, the N-terminal tags were cleaved off by TEV-protease digestion. Constructs containing a DBD were further purified using heparin affinity chromatography. Other domains were subjected to a Ni-affinity chromatography again. For full-length KLF4, gel filtration chromatography was done as a final step using a Superdex 200 16/60 preparative gel filtration column (GE Healthcare). Protein purity of >95% was determined by SDS-gel electrophoresis. Labelling of KLF4-FlAsH was done as described before .

## DNA constructs and production

5’ labelled DNA (Supplementary Table S1) was either purchased with an Alexa488 fluorophore (*P, Operon) or fluorescein (*PK, *P5K, *P10K, *P30K, *K, *47K, Sigma or IDT) and annealed to double-stranded DNA prior to use. For *P88K, *P185K, *P1000K, and*108K, we constructed a plasmid containing approximately 1000 base pairs of the *p21* promoter with the p53 RE at the centre. This plasmid and *P as forward primer were used to produce labelled DNA in sufficient quantities by performing several PCR reactions using FastStart PCR Master (Roche). Reverse primers were designed to guarantee appropriate sequence length and to include a KLF4 RE. Samples were purified using a PCR purification kit (QIAGEN) and purity was controlled by agarose gel electrophoresis.

## References

1. Nikolova PV, Henckel J, Lane DP, Fersht AR (1998) Semirational design of active tumor suppressor p53 DNA binding domain with enhanced stability. Proc Natl Acad Sci U S A 95: 14675-14680.

2. Brandt T, Petrovich M, Joerger AC, Veprintsev DB (2009) Conservation of DNA-binding specificity and oligomerisation properties within the p53 family. BMC Genomics 10: 628.

3. Joerger AC, Allen MD, Fersht AR (2004) Crystal structure of a superstable mutant of human p53 core domain. Insights into the mechanism of rescuing oncogenic mutations. J Biol Chem 279: 1291-1296.

4. Tidow H, Melero R, Mylonas E, Freund SM, Grossmann JG, et al. (2007) Quaternary structures of tumor suppressor p53 and a specific p53 DNA complex. Proc Natl Acad Sci U S A 104: 12324-12329.

5. Veprintsev DB, Fersht AR (2008) Algorithm for prediction of tumour suppressor p53 affinity for binding sites in DNA. Nucleic Acids Res 36: 1589-1598.

6. Veprintsev DB, Freund SM, Andreeva A, Rutledge SE, Tidow H, et al. (2006) Core domain interactions in full-length p53 in solution. Proc Natl Acad Sci U S A 103: 2115-2119.

7. Hipps DS, Packman LC, Allen MD, Fuller C, Sakaguchi K, et al. (1994) The peripheral subunit-binding domain of the dihydrolipoyl acetyltransferase component of the pyruvate dehydrogenase complex of Bacillus stearothermophilus: preparation and characterization of its binding to the dihydrolipoyl dehydrogenase component. Biochem J 297 ( Pt 1): 137-143.

8. Adams SR, Campbell RE, Gross LA, Martin BR, Walkup GK, et al. (2002) New biarsenical ligands and tetracysteine motifs for protein labeling in vitro and in vivo: synthesis and biological applications. J Am Chem Soc 124: 6063-6076.

9. Joerger AC, Ang HC, Fersht AR (2006) Structural basis for understanding oncogenic p53 mutations and designing rescue drugs. Proc Natl Acad Sci U S A 103: 15056-15061.
