# Supplementary material for: Molecular Basis for Modulation of the p53 Target Selectivity by KLF4
Source: PLoS One. 2012 Oct 30;7(10):e48252. doi: 10.1371/journal.pone.0048252 (PMC3484126; doi:10.1371/journal.pone.0048252)
Supplement: Text S2 — Supplementary results section on the in vitro characterisation of KLF4. (DOC) [file pone.0048252.s015.doc]

## Supplementary results

### Recombinant KLF4 is a monomeric protein and binds DNA very selectively

In order to study the putative p53-KLF4 interaction *in vitro* using biophysical methods, we expressed and purified KLF4 (isoform 2, 1-479) and p53 in *Escherichia coli* cells. We did sedimentation velocity analytical ultracentrifugation (SV-AUC) experiments to determine the oligomerisation state of KLF4. The sedimentation trace obtained by SV-AUC showed only one peak with a sedimentation coefficient of S = 1.3, corresponding to monomeric KLF4 (Supplementary Figure S1A).

Secondly, we wanted to assess the DNA-binding properties of KLF4 using fluorescence anisotropy titrations. A minimal binding sequence RRGGYGY (used for *K-2) has been reported . More recently a refined consensus sequence has been obtained using a genome-wide chromatin immuno-precipitation (ChIP) approach (GGGTGTGGCC, used for *K-1, ). These sequences were templates for the design of fluorescein-labelled DNA (Table S1) which was used for fluorescence anisotropy titrations. We were able to detect sub-nanomolar to nanomolar binding of KLF4 to *K-1 depending on the ionic strength (Table S2, Figure S1C). Furthermore, DNA binding is very specific and *K-2 was bound 100-fold weaker by KLF4, whereas binding to the p53 RE was completely abolished (Table S2, Figure S1C). All observed binding events were non-cooperative.

Further, we wanted to determine the stoichiometry of the KLF4-DNA complex. We used fluorescence detection SV-AUC (FDSV-AUC) experiments with fluorescently labelled DNA (*K-1) and KLF4 in 20-fold excess. The sedimentation coefficient distribution showed one peak at *S* = 1.5, most likely corresponding to a 1:1 complex (Figure S1B). No complete sedimentation of isolated DNA was observed at the rotor speed used.

### References

1. Shields JM, Yang VW (1998) Identification of the DNA sequence that interacts with the gut-enriched Kruppel-like factor. Nucleic Acids Res 26: 796-802.

2. Chen X, Xu H, Yuan P, Fang F, Huss M, et al. (2008) Integration of external signaling pathways with the core transcriptional network in embryonic stem cells. Cell 133: 1106-1117.
